# Identification and Characterization of RBM44 as a Novel Intercellular Bridge Protein

**DOI:** 10.1371/journal.pone.0017066

**Published:** 2011-02-25

**Authors:** Tokuko Iwamori, Yi-Nan Lin, Lang Ma, Naoki Iwamori, Martin M. Matzuk

**Affiliations:** 1 Department of Pathology and Immunology, Baylor College of Medicine, Houston, Texas, United States of America; 2 Department of Molecular and Cellular Biology, Baylor College of Medicine, Houston, Texas, United States of America; 3 Department of Molecular and Human Genetics, Baylor College of Medicine, Houston, Texas, United States of America; Texas A&M University, United States of America

## Abstract

Intercellular bridges are evolutionarily conserved structures that connect differentiating germ cells. We previously reported the identification of TEX14 as the first essential intercellular bridge protein, the demonstration that intercellular bridges are required for male fertility, and the finding that intercellular bridges utilize components of the cytokinesis machinery to form. Herein, we report the identification of RNA binding motif protein 44 (RBM44) as a novel germ cell intercellular bridge protein. RBM44 was identified by proteomic analysis after intercellular bridge enrichment using TEX14 as a marker protein. RBM44 is highly conserved between mouse and human and contains an RNA recognition motif of unknown function. RBM44 mRNA is enriched in testis, and immunofluorescence confirms that RBM44 is an intercellular bridge component. However, RBM44 only partially localizes to TEX14-positive intercellular bridges. RBM44 is expressed most highly in pachytene and secondary spermatocytes, but disappears abruptly in spermatids. We discovered that RBM44 interacts with itself and TEX14 using yeast two-hybrid, mammalian two-hybrid, and immunoprecipitation. To define the *in vivo* function of RBM44, we generated a targeted deletion of *Rbm44* in mice. *Rbm44* null male mice produce somewhat increased sperm, and show enhanced fertility of unknown etiology. Thus, although RBM44 localizes to intercellular bridges during meiosis, RBM44 is not required for fertility in contrast to TEX14.

## Introduction

Cytokinesis in somatic cells concludes with the formation of a midbody, which is abscised to form individual daughter cells [1]. In contrast, cytokinesis in differentiating germ cells transforms the midbody into a permanent intercellular bridge interconnecting daughter cells through a large cytoplasmic channel [2]. Several roles have been proposed about the functions of mammalian intercellular bridges for spermatogenesis [3]. The proposed roles for the intercellular bridges include sharing of essential signals among interconnected germ cells, synchronization of germ cell divisions [4], [5], [6], and chromosome dosage compensation in haploid cells after meiosis [4], [7], [8]. There is also biochemical evidence that mRNA produced in a subset of cells of a clone will be expressed in all cells of the clone [7].

Previously, we identified testis-expressed gene 14 (TEX14) as a intercellular bridge protein in germ cells [9], [10]. TEX14 localizes to all intercellular bridges in the testis and therefore functions as a marker protein of intercellular bridges. In the absence of TEX14 *in vivo*, spermatogenesis failed at the spermatocyte stage because of the disruption of intercellular bridges, resulting in sterility in male mice [9]. Thus, TEX14 is essential for the formation of intercellular bridge, spermatogenesis, and fertility in male mice [9]. These studies provide the first evidence that intercellular bridges are essential for spermatogenesis and fertility. Recently, we refined the mechanism of intercellular bridge formation in germ cells and found that TEX14 interacts with centrosomal protein 55 kDa (CEP55) to form intercellular bridges [11]. CEP55 localizes to the midbody in somatic cells and functions as a key protein for abscission with ALIX (ALG-2 interacting protein X) and TSG101 [a component of the ESCRT-1 (endosomal sorting complex required for transport-1) complex], which are the direct downstream proteins of CEP55 [12], [13]. We identified CEP55 as a stable intercellular bridge protein in the testis and ovary, and TEX14 binds to CEP55 to inhibit CEP55:ALIX and CEP55:TSG101 interactions [11]. The exogenous expression of TEX14 inhibits entry of ALIX into the midbody resulting in the formation of several interconnected somatic cells because of failure to complete cytokinesis. In addition, TEX14 binds with mitotic kinesis-like protein 1 (MKLP1), which is one of the centralspindlin complex proteins that localizes to the midbody during cytokinesis [14]. Because CEP55 also binds MKLP1 to complete cytokinesis in somatic cells [15], we believe that TEX14 binds both MKLP1 and CEP55 and “locks” the complex in an stable configuration so that midbodies are transformed into intercellular bridges, thereby preventing abscission.

Sharing of mRNAs across interconnected germ cells are potentially critical for spermatogenesis and RNA binding proteins are involved in various aspects of RNA metabolism including RNA stability, RNA processing, and translation [16]. A subset of RNA binding proteins form a family that is identified by one or more copies of an 80–90 amino acid RNA recognition motif (RRM), that has been shown to mediate RNA binding proteins [16], [17]. Several RNA binding proteins have been shown to play roles in the testis including Nanos2 [18], Nanos3 [18], TIAR [19], MSY2 [20]. TLS [21], translin [22], DAZL[23], and Boule [24]. Herein, we report the identification and targeted ablation of a novel RRM-containing intercellular bridge protein, RBM44.

## Materials and Methods

### Enrichment of intercellular bridges

Intercellular bridge preparations were obtained from eight-week-old wild-type mice testes as previously described [14]. The enriched intercellular bridge fraction (referred to as PT) was transferred to Superfrost^^®reg;^^/Plus Microscope Slides (Fisher Scientific, Houston, TX) and allowed to air dry. After drying, the slides were lightly rinsed in TBS (100 mM Tris–HCl, pH 7.5; 0.9% 150 mM NaCl), and used for immunofluorescence to detect mouse RBM44 and TEX14 as described below.

### Generation of anti-RBM44 antibody

**Figure 1:**
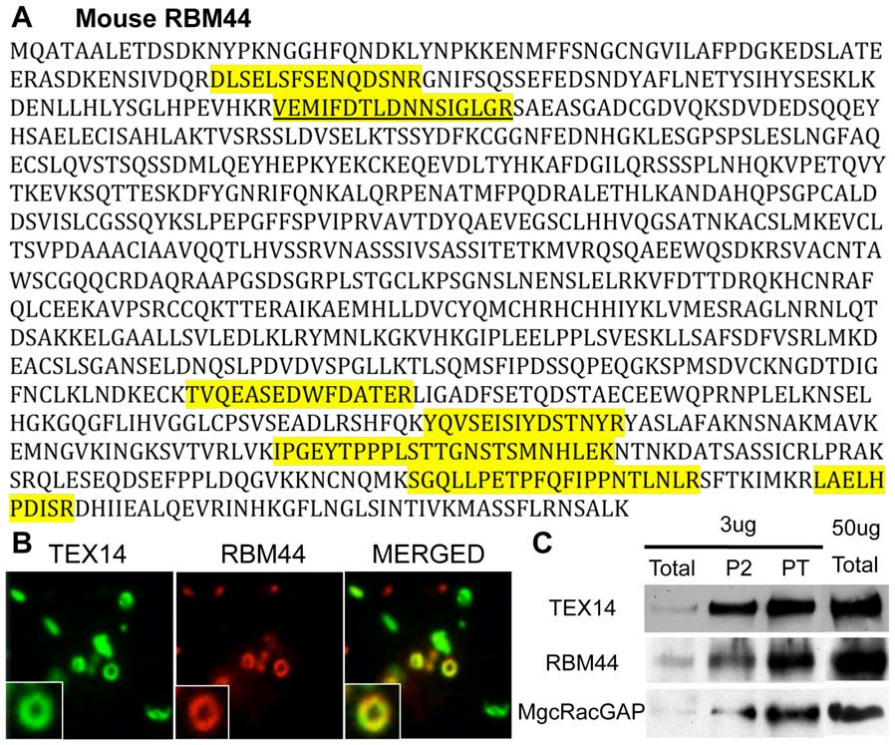
Identification of intercellular bridge protein, RBM44. A, RBM44 peptides that were identified by proteomic analysis: the identified peptides are highlighted in yellow and two overlapping peptides are underlined. B, Immunofluorescence of the isolated intercellular bridge fraction from eight-week-old wild-type mouse testes: green, TEX14; red, RBM44; yellow, merged. C, Western blot analyses of the enriched total, P2, and PT functions, using anti-TEX14, anti-RBM44, and anti-MgcRacGAP antibodies.

**Figure 2:**
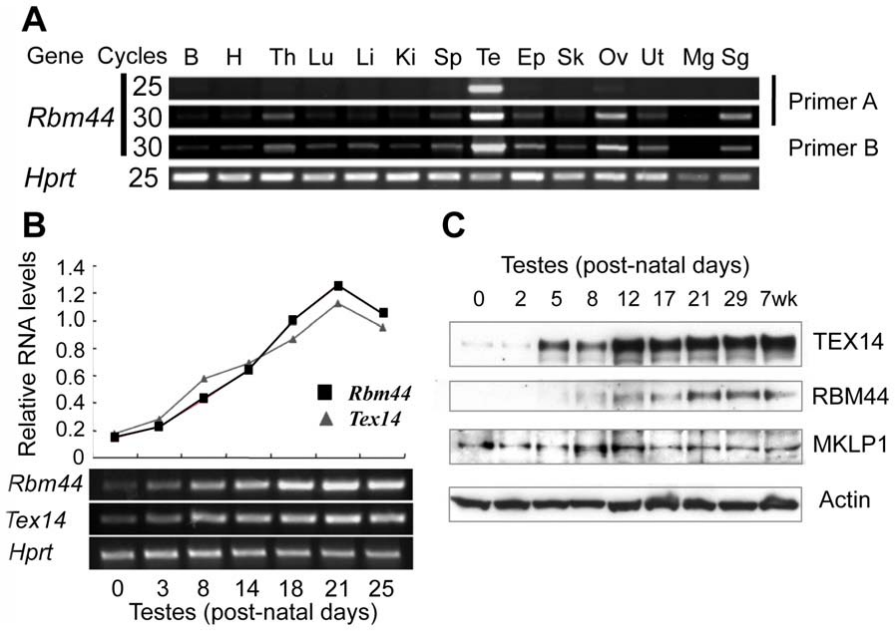
Tissue distribution and developmental expression of RBM44 in the testes. A, Multi-tissue RT-PCR using two primer sets for *Rbm44* (primer A and B) and *Hprt* for a control. PCR cycles are 25 or 30. [B: brain, H: heart, Th: thymus, Lu: lung, Li: liver, Ki: kidney, Sp: spleen, Te: testis, Ep: epididymis, Sk: skeletal muscle, Ov: ovary, Ut: uterus, Mg: mammary gland, Sg: submandibular gland.]. B, RT-PCR comparison of *Rbm44* and *Tex14* expression in different developmental stages of mouse testes. The graphs show relative ratios of *Rbm44*/*Hprt* and *Tex14*/*Hprt*. C, Western blot analyses show the protein expression pattern and protein levels of TEX14, RBM44, MKLP1, and ACTIN in different developmental stages of mouse testes.

**Figure 3:**
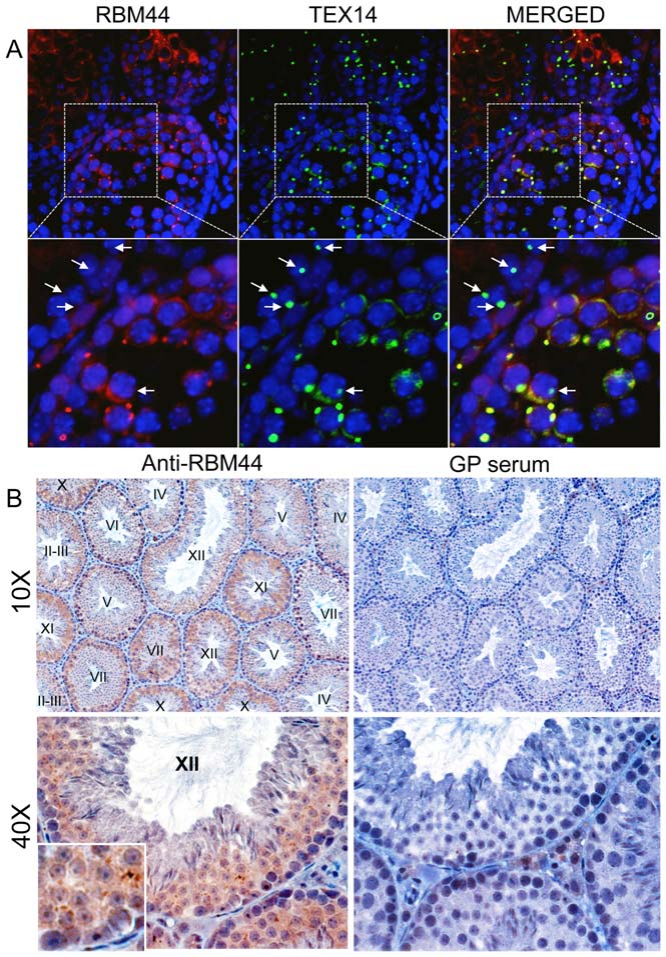
RBM44 localization in the testis. A, RBM44 co-localizes with TEX14 in the testis. Immunofluorescence in post-natal day 18 mouse testis using anti-TEX14 and anti-RBM44 antibodies: red, RBM44; green, TEX14; blue, DAPI; yellow, merged; arrows, TEX14-positive and RBM44-negative intercellular bridges. High-magnification images are derived from the boxed regions, respectively. B, RBM44 also localizes to both the cytoplasm and intercellular bridges. Immunohistochemistry was performed on 3-month-old mouse testes using anti-RBM44 antibody (left) and guinea pig serum as a control (right).

**Figure 4:**
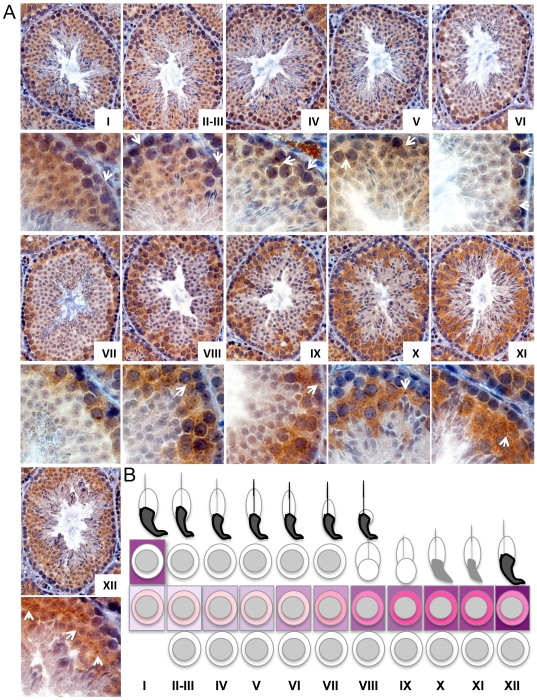
RBM44 localizes in the cytoplasm and intercellular bridges at specific time points. A, Immunohistochemistry of 3-month-old mouse testes using anti-RBM44 antibody was examined by the staging of spermatogenesis in comparison with PAS staining in serial sections. The arrows denote examples of RBM44-positive intercellular bridges. B, Summary of RBM44 expression pattern in spermatogenesis. The intensity of RBM44 cytoplasmic staining is depicted diagrammatically in shades of pink. The degree of RBM44 localization to intercellular bridges is shown similarly in purple bars. Peak staining of intercellular bridges occurs in stage XII, while cytoplasmic staining is highest prior in stages IX-XI.

**Figure 5:**
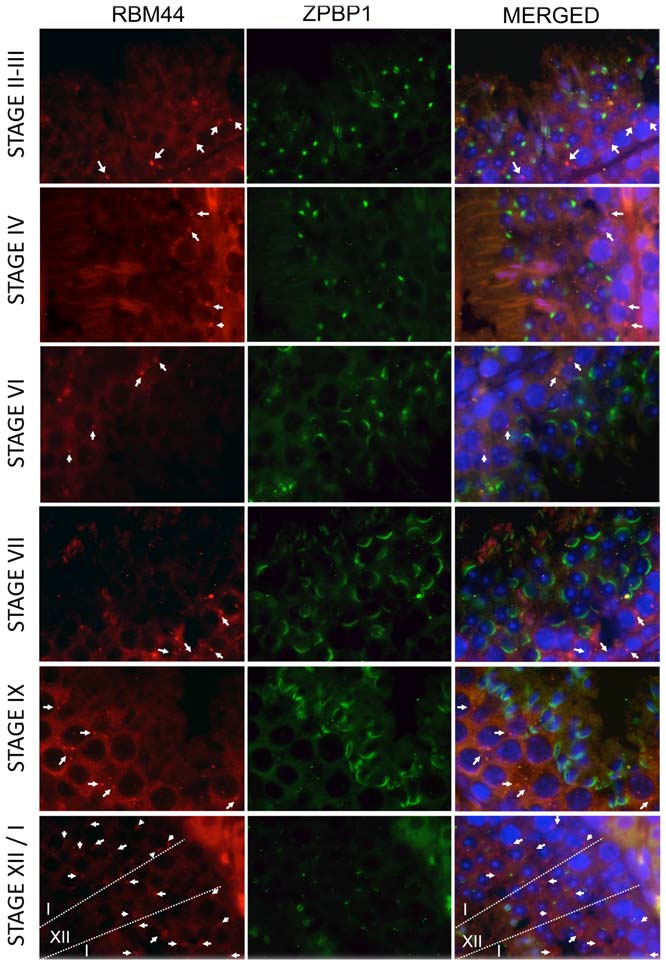
RBM44 localizes to the cytoplasm and intercellular bridges in pachytene spermatocytes through secondary spermatocytes. Immunofluorescence in 3-month-old mouse testis using anti-RBM44 and anti-ZPBP1 antibodies: red, RBM44; green, ZPBP1; blue, DAPI; yellow, merged; arrows, RBM44-positive intercellular bridges.

To determine the spatiotemporal expression of RBM44, we generated antibodies to independent regions of RBM44. One antibody was generated against N-terminal amino acids 471–609 and the other antibody to C-terminal amino acids 740–1013 of the mouse RBM44 protein that contained the RRM domain (amino acids 793–860). Both peptides were used to generate polyclonal antibodies in guinea pigs using methods described previously [9]. The antibodies were affinity purified with the RBM44 antigens using the AminoLink Plus Immobilization Kit (Pierce, Rockford, IL). The antibody to amino acids 740–1013 of mouse RBM44 was used in Figures 1, 2, 3, 4, and 5. The antibody to N-terminal amino acids 740–1013 of mouse RBM44 was used in Figures 8 and 9 to confirm that our mutation generated a null mutation.

### Immunofluorescence and immunohistochemistry analyses

Mouse testes were fixed overnight at 4°C within 4% paraformaldehyde in TBS, followed by subsequent incubation overnight at 4°C in 70% ethanol. The testes were processed and embedded by the Department of Pathology Core Services Laboratory (Baylor College of Medicine, Houston, TX), and 4 µm micrometer sections were cut and prepared for immunostaining. Samples were blocked in 3 or 5% BSA/TBS blocking buffer for 1 h at room temperature, and then incubated with affinity purified primary antibodies/blocking buffer overnight at 4°C using the following dilutions: goat or rabbit anti-TEX14 antibody, 1∶500; guinea pig anti-RBM44 antibody, 1∶200 (IF) or 1∶50 (IC); guinea pig anti-CEP55 antibody, 1∶500; and goat anti-ZPBP1 antibody, 1∶1000. For immunofluorescence, Alexa 488 and Alexa 594 conjugated secondary antibodies were purchased from Invitrogen (Carlsbad, CA). Samples were mounted with VECTASHIELD mounting media with DAPI (Vector, Burlingame, CA) and sealed with coverslips (VWR Scientific, Westchester, PA). For immunohistochemistry, the secondary antibody was biotinylated goat anti-guinea pig IgG (H+L) (Vector, 1∶500 dilution). VECTASTAIN ABC system and DAB (Vector) steps were performed according to the manufacturer's instructions. Samples were examined under a fluorescence microscope Axiovert 200µ (Carl Zeiss MicroImaging, Thornwood, NY). Fluorescence and differential interference contrast (DIC) images were captured and processed using AxioVision Rel 4.6.

### RT-PCR

Total RNAs extracted from tissue samples were reverse transcribed into cDNAs using SuperScript™ II Reverse Transcriptase (Invitrogen). PCR was performed by following primer sets: *Rbm44* primer A-forward, 5′-CGTCCAGGAAGCAAGTGAAGA; *RBM44* primer A-reverse, 5′-TTTCAAAGCAGAGTTTCTCAG; *Rbm44* primer B-forward, 5′-GCATGCTCGCTGATGAAAGA; *Rbm44* primer B-reverse, 5′- CCTCTCAGTGGTCTTCTGAC; *Tex14*-forward, 5′-GGGATGCTTCATTAAAGTTTGC; *Tex14*-reverse, 5′-ATTTCAAGTGTGCCTCTCCATT; *Hprt*-forward, 5′-CATCACATTGTGGCCCTCTG; *Hprt*-reverse, 5′- CCTTAACCATTTTGGGGCTGT.

### Generation of N-terminal flag-tagged Tex14 or Rbm44 and myc-tagged Rbm44 constructs

Mouse *Tex14* of sequence was ligated into pCMV-tag2 vector (Stratagene, La Jolla, CA), which contains an N-terminal flag tag sequence. The open reading frame (ORF) of the mouse *Rbm44* was cloned from testis cDNA and subcloned into pcDNA3 vector containing an N-terminal flag or myc tag sequence. Purified plasmid DNA was obtained using the QIAprep^®reg;^ Spin Miniprep Kit (QIAGEN Sciences, Germantown, MD), and all constructs were sequenced for integrity.

### Cell culture and transfection

HEK293T cells (human embryonic kidney 293 cells with expression of SV-40 large T antigen; provided by Tissue culture core at Baylor College of Medicine) were maintained in DMEM (Invitrogen) medium supplemented with 10% fetal calf serum (SAFC Biosciences, Lenexa, KS), 1% L-glutamine (Invitrogen), and penicillin-streptomycin (Invitrogen) and grown on Poly-D-Lysine-coated cover slips (Sigma, St. Louis, MO) in culture plates at 37C° in a humidified 5% CO_2_ atmosphere. For immunoprecipitation and immunoblotting experiments, cells were seeded at 50–80% confluence in 10 cm^2^ dishes (Corning, Corning, NY) and transiently transfected using Fugene^®reg;^ 6 or HD Transfection Reagent (Roche, Mannheim, Germany) according to the manufacturer's instructions.

### Co-immunoprecipitation and Western blot analysis

The flag-tagged *Tex14* and *Rbm44* vectors were co-transfected with the myc-tagged *Rbm44* vector in HEK293T cells, and followed by immunoprecipitation using anti-FLAG or anti-MYC antibodies and western blot using anti-FLAG anti-MYC antibodies as previously described [11]. The immunoprecipitates, total cell lysates, and intercellular bridge preparations (referred to as Total, P2 and PT) were separated by 3–8% Tris-Acetate gel (Invitrogen) and transferred onto a nitrocellulose membrane (Protran BA83, Whatman GmbH, Germany). Western blot assay was performed using mouse anti-MYC monoclonal antibody (1∶5000; BD Biosciences, San Jose, CA), mouse anti-FLAG monoclonal antibody (1∶8000; SIGMA), goat anti-TEX14 antibody, guinea pig anti-RBM44 antibody, rabbit anti-MgcRacGAP antibody, guinea pig anti-MKLP1 antibody, mouse anti-Actin antibody (1–2µg/ml) as a primary antibodies and horseradish peroxidase-conjugated anti-mouse, goat, rabbit, and guinea pig IgG (1∶10000; Jackson Immunoresearch, West Grove, PA) as a secondary antibody. Proteins were detected with chemiluminescence by SuperSignal^®reg;^ West Pico Chemiluminescent Substrate (Thermo Scientific, Rockford, IL) and exposed to BioMax XAR film (Eastman Kodak, Rochester, NY).

### Yeast Two- Hybrid System and oxygen-biosensor assay

Protein-protein interactions were evaluated using the Matchmaker™ Two- Hybrid System 3 (Clontech, Mountain View, CA) as described previously [11], [14]. Mouse full-length *Tex14* and *Mklp1* were previously subcloned into the Matchmaker *GAL4* two-hybrid pGBKT7 and pGADT7 vectors [14]. The full-length mouse *Rbm44* construct was made using the Matchmaker *GAL4* two-hybrid pGBKT7 bait vector and pGADT7 prey vectors. Some results of Y2H interactions were examined with an oxygen-biosensor assay that measured the fluorescence emitted by an oxygen-sensing platform that measures yeast growth in the selective medium (Clontech).

### Mammalian Two-Hybrid System

The CheckMate™/Flexi^®reg;^ Vector Mammalian Two- Hybrid System (Promega, Madison, WI) was used for studying the interactions of proteins as previously described [11]. Mammalian two-hybrid pACT and pBIND vectors, which express VP16-RBM44 or GAL4-RBM44 fusion proteins respectively, were constructed and cotransfected with pGL4Cherry [mCherry/GAL4UAS/Hygro] vector [11] in HEK293T cells using FuGENE HD Transfection Reagent. pBIND vector expresses *Renilla* Luciferase by separate promoter also. The transfected cells were examined forty-four hours later for red fluorescence of cells using a microscope (Axiovert 40 CFL, ZEISS). AxioVision Rel 4.6 was used for the analysis of image. The combination of empty pACT and pBIND (GAL4-RBM44) vectors served as a negative control. The cells were lysed using *Renilla* Luciferase Assay Reagent (Promega), and red fluorescence and *Renilla* Luciferase were measured using a POLAR Star Omega microplate reader (BMG LabTech, Offenburg, Germany); red fluorescence, excitation/emission = 585/620-10. Protein interactions were quantified using the ratio of red fluorescence to *Renilla* Luciferase.

### Alignment of the motif and flanking sequences from RBM44 orthologs

The predicted *Rbm44* cDNAs or proteins were analyzed using following database software; UCSC Genome Bioinformatics (http://genome.ucsc.edu/), Multalin (http://multalin.toulouse.inra.fr/multalin/multalin.html), and SMART (http://smart.embl-heidelberg.de/). *RBM44* cDNAs were cloned using 5′ and 3′ rapid amplification of cDNA ends by the SMART RACE cDNA amplification kit (BD Biosciences). The ORFs of deduced and cloned cDNAs were determined using the EditSeq program of DNASTAR software (Madison, WI).

### Construction of the Rbm44 targeting vector

A targeting construct was generated using a recombineering strategy [25], [26]. Briefly, 8.1 kb of genomic region containing exons from 11 to 13 of *Rbm44*, which include RRM domain, was retrieved from BAC bMQ 158A18 [27](Welcome Trust Sanger Institute) into pBluescript SK containing diphtheria toxin A for negative selection (pDTA.3 kindly provided by Dr. Pumin Zhang). Exons 11 and 12 of *Rbm44* were franked by a *frt-pgkNeo-frt-loxP* cassette and a *loxP* sequence. The linearized targeting construct was electroporated into AB2.1 embryonic stem (ES) cells, which are derived from 129S7/SvEv strain mice. ES cell clones were selected in M15 supplemented with 0.18 mg/ml G418. Targeted clones were screened by Southern blot analysis using 5′ and 3′ probes. Two of the correctly targeted clones were expanded and injected into C57BL/6J blastocysts. Males chimeric for the *Rbm44* ^tm1Zuk^ (*Rbm44* ^(frt-neo-frt)-loxP^) allele were bred to *EIIA-Cre* female mice to produce mice with Cre recombination of the loxP sites and deletion of exons 11 and 12 (i.e., *Rbm44* heterozygous mutant (*Rbm44* ^+/−^) mice). Male and female *Rbm44* ^+/−^ mice intercrossed to obtain homozygous mutant mice (*Rbm44^−/−^*). Mice were genotyped by Southern blot using the probe indicated in Figure 8A or PCR analysis using the following primers: *Rbm44* wt allele-forward, 5′-ACTGCATGGAAAAGGTCAGG; *Rbm44* wt allele-reverse, 5′- GGAGTGGCTCCTGCTTACTG; *Rbm44* deleted allele-forward, 5′- AACAGTCAGAGCCCCCTTTA; *Rbm44* deleted allele-reverse: 5′-ACCATGGAGGCTATGTGTGA. All mice were maintained on a C57BL/6J/129S7/SvEv hybrid genetic background; littermates including wild-type controls were used in these experiments. All experimental animals were maintained in accordance with the National Institutes of Health Guide for the Care and Use of Laboratory Animals. This study was approved by the institutional animal care and use committee at the Baylor College of Medicine (approval number AN-716).

### Sperm count

Total epididymal sperm counts were performed as described in Roy *et al* [28]. Caudal epididymides were dissected from 3-month-old wild-type, *Rbm44* heterozygous, and null mice (n = 3–5 mice/genotype). Caudal epididymides were dissected and minced in 1 ml pre-warmed M16 medium (Sigma–Aldrich, St. Louis, MO), and incubated at 37°C with 5% CO_2_ for two hours so that sperm were allowed to swim into the medium. Sperm were diluted in water to cause immobility prior to counting with a hemocytometer. Sperm were counted 4 times for each sample and then averaged.

### Statistical analysis

Data were analyzed by analysis of variance according to the Student's *t*-test. Differences between the mean values were considered to be statistically significant at p<0.05.

## Results

### Identification of a novel intercellular bridge protein

To further understand the characteristics and functions of intercellular bridges, we used a previously developed biochemical method to enrich intercellular bridges from mouse testes to identify new intercellular bridge proteins [14]. This enriched preparation was subsequently examined by LC/MS/MS proteomic analysis [14]. In addition to TEX14, which is a marker of intercellular bridge enrichment, we identified 19 proteins that have roles in cytokinesis including MKLP1, MgcRacGAP [14], and CEP55 [11]. Furthermore, eight of the peptides in our bridge preparation matched the hypothetical protein XP_287014 (Figure 1A). XP_287014 contains an RNA recognition motif (amino acids 793-861) and is now annotated as RNA binding motif protein (RBM44). To confirm that XP_287014 is a component of the intercellular bridge and not a contaminant, we generated an antibody against the 274 unique amino acids in the C-terminus of XP_287014 and performed immunofluorescence and western blot analysis using the enriched intercellular bridges from eight-week-old mouse testes (Figure 1B). We discovered that RBM44 co-localized with TEX14 in the partial rings of some but not all of the purified intercellular bridges (Figure 1B), and biochemically co-enriched similar to TEX14 and MgcRacGAP (Figure 1C). Thus, in addition to TEX14 (the first essential intercellular bridge protein [9]), the cytokinesis proteins MKLP1 and MgcRacGap [14], and the abscission protein CEP55 [11], RBM44 is the latest novel protein intercellular bridge protein that we have discovered.

### Expression pattern of RBM44

To identify the mouse tissues that express *Rbm44*, we performed RT-PCR using cDNAs from multiple tissues. *Rbm44* is expressed highly in testis but was detectable in other tissues after more cycles of PCR amplification (Figure 2A). We further examined the expression pattern of RBM44 in post-natal testes. The *Rbm44* expression pattern was similar to *Tex14* (Figure 2B), and western blot analysis detected RBM44 expression in mouse testes beginning at post-natal day 5, which is later than our findings for TEX14 and MKLP1 (Figure 2C). Thus, RBM44 is hypothesized to not function in initial intercellular bridge formation but as a secondary component of intracellular bridges at later time points.

### RBM44 is localized in the cytoplasm and intercellular bridges from pachytene to secondary spermatocyte stages

To analyze the localization of RBM44, we performed immunofluorescence using anti-RBM44 and anti-TEX14 antibodies, and immunohistochemistry using the anti-RBM44 antibody. Consistent with the immunofluorescence using purified intercellular bridges (Figure 1B), RBM44 co-localizes with TEX14 in intercellular bridges as small ring-shaped intercellular bridge structures in germ cells but not in all intercellular bridges (Figure 3A; see Figure 9 described later). RBM44 is also localized to the cytoplasm in addition to the intercelllar bridges, and the localization and signal intensities are dependent on the stage of spermatogenesis (Figure 3B). Specific tubules show strong RBM44 expression in the cytoplasm, and many RBM44-positive intercellular bridges are present with a peak in stage XII (Figure 3B and 4).

To further define the localization of RBM44, we performed immunofluorescence using anti-RBM44 and anti-ZPBP1 antibodies and PAS staining with serial sections, and identified the staging by the formation of the acrosome (Figure 5 and S3A–C). RBM44 positive intercellular bridges were identified in all stages of tubules but dynamically increased from stage I pachytene spermatocytes to stage XII secondary spermatocytes and disappeared after the formation of step 1 round spermatids (stage I) (Figure 4A, B, 5 and S3). In addition, RBM44 is localized in the cytoplasm in pachytene spermatocytes to secondary spermatocytes (Figure 4A, B, 5 and S3). The cytoplasmic signals also increased dramatically in stages X-XII, decrease from step 1 to step 3 spermatids, and disappear in step 4 spermatids (Figure 4A, B and S3).

### RBM44 interacts with itself and TEX14

**Figure 6:**
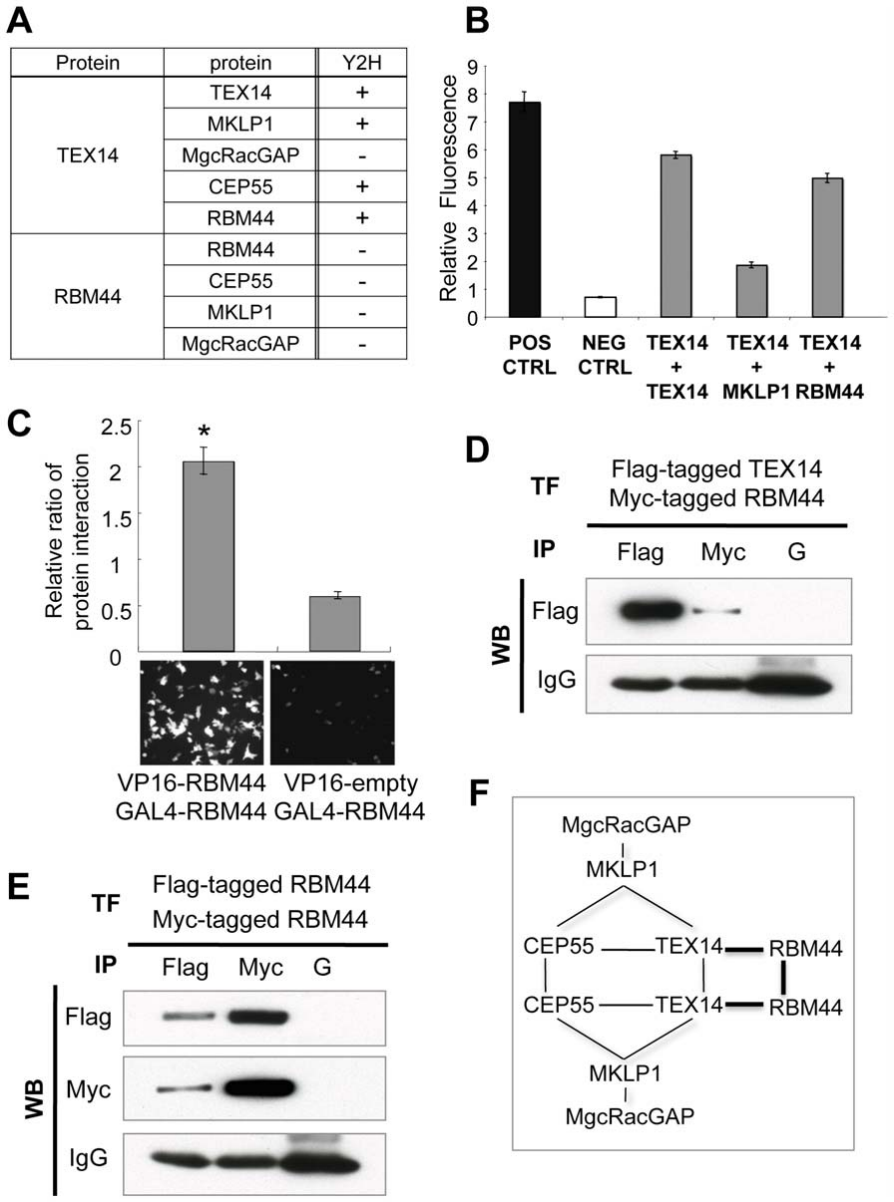
RBM44 interacts with itself and TEX14. A, Yeast two-hybrid assay using vectors encoding full-length mouse *Rbm44*, *Tex14*, *Mklp1*, *MgcRacGap*, and *Cep55*; +, interaction; -, no interaction. B, The relative ratios of TEX14-TEX14, TEX14-MKLP1 and TEX14-RBM44 interactions were determined using a oxygen-biosensor system. C, Mammalian two-hybrid assay indicates RBM44 self-interaction. The relative ratio of red fluorescence divided by *Renilla* luminescence is shown (top). VP16-empty vector is a control. Red fluorescence is visualized under the fluorescence microscope in the mammalian two-hybrid assay using VP16-RBM44 and GAL4-RBM44 (bottom left panel), but not in the control experiment using VP16-empty and GAL4-RBM44 vectors (bottom right panel). D, The *Flag*-*Tex14* and *Myc-Rbm44* vectors were cotransfected in HEK293T cells, and followed by immunoprecipitation (IP) with anti-FLAG or anti-MYC antibodies and western blot analysis using the anti-FLAG antibody as shown. Protein G (“G”) is a control. E, The Flag-tagged *Rbm44* and Myc-tagged *Rbm44* were overexpressed in HEK293T cells, and immunoprecipitation and western blot analyses using anti-FLAG or anti-MYC antibodies were performed. F, Model of the interaction between RBM44 and the other proteins in the intercellular bridge.

Previously, we reported that full-length TEX14 interacts with itself, MKLP1, and CEP55 using yeast two-hybrid assay (Figure 6A and B) [11], [14]. Herein, we showed that the full-length RBM44 interacts with the full-length TEX14 but not with MKLP1 and MgcRacGAP using yeast two-hybrid assays (Figure 6A and B). Furthermore, full-length RBM44 was shown to interact with itself using mammalian two-hybrid assays (Figure 6C, graph and bottom panel), although yeast two-hybrid assays show no interaction between RBM44 and itself (Figure 6A). After FLAG-tagged full-length *Tex14* and MYC-tagged full-length *Rbm44* vectors were co-transfected in HEK293T cells and followed by immunoprecipitation, FLAG-TEX14 and MYC-RBM44 proteins were shown to interact (Figure 6D). Additionally, after a FLAG-tagged *Rbm44* vector was co-transfected with a MYC-tagged *Rbm44* vector into HEK293T cells, immunoprecipitation results also showed FLAG-RBM44 and MYC-RBM44 interact (Figure 6E). Thus, combined with our previous findings [11], [14], RBM44 forms a complex with core intercellular bridge proteins (Figure 6F).

### RBM44 is highly conserved in multiple species

**Figure 7:**
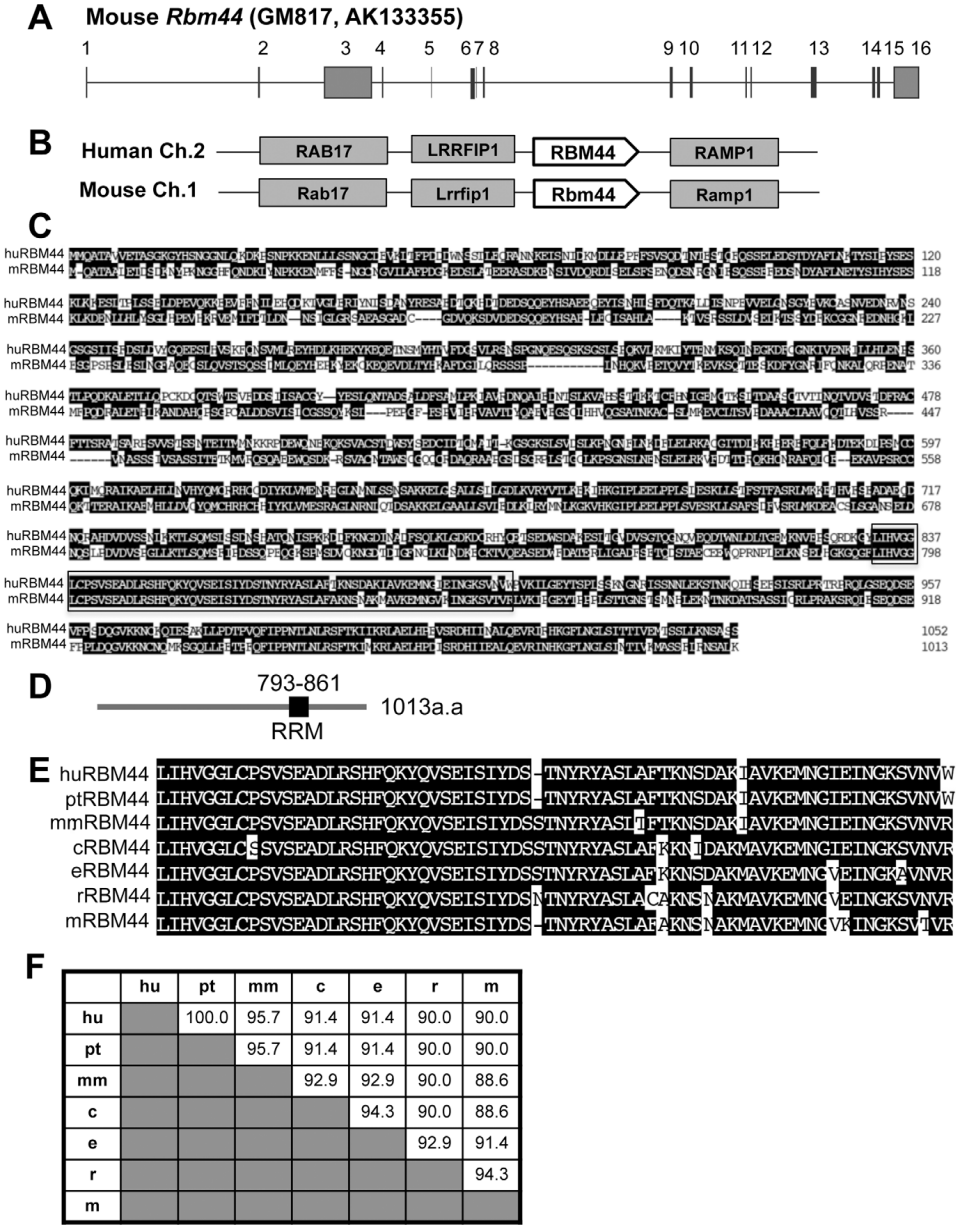
Evolutionary comparison of mammalian RBM44 genes and their encoded proteins. A. Genomic structure of mouse *Rbm44* with exons numbered. B, The genomic localization of RBM44 (identified as RBM44). C, The consensus sequence between the predicted human and mouse RBM44 protein sequences: box, RRM domain. D, Location of the RRM domain in mouse RBM44. E-F, Alignment of RRM amino acid sequences among seven mammals (E), and their percent homology (F). [h: *Homo sapiens* (human), p: *Pan troglodytes* (chimpanzee), mm: *Macaca mulatta* (rhesus macaque), c; *Canis famliliaris* (dog), e: *Equus caballus* (horse), r: *Rattus rattus* (rat), m: *Mus musculus* (mouse)].

Using 5′ and 3′ RACE PCR (Figure 7A), we confirmed that the mouse *Rbm44* gene (geneID_329207) is encoded by 16 exons. Mouse *Rbm44* is located on chromosome 1 between *Lrrfip1* and *Ramp1*, and human RBM44 is at a syntenic position on chromosome 2 (Figure 7B). The *RBM44* mRNAs encode 1013 aa and 1052 aa proteins in mouse and human, respectively (Figure 7C and D), and these human and mouse proteins are highly conserved (Figure 7C). The amino acid alignment between human and mouse sequences shows 55% identity. RBM44 has a RRM domain, a single main motif that is localized at amino acids 793–861 in the 1013aa mouse RBM44 (and amino acids 832–900 in the 1052aa human RBM44) (Figure 7C and D). The RRM domain shows 96.7% homology between human and mouse (Figure 7E and F). The RRM domain of RBM44 is highly conserved among other species (Figure 7E and F).

### Targeted deletion of Rbm44

**Figure 8:**
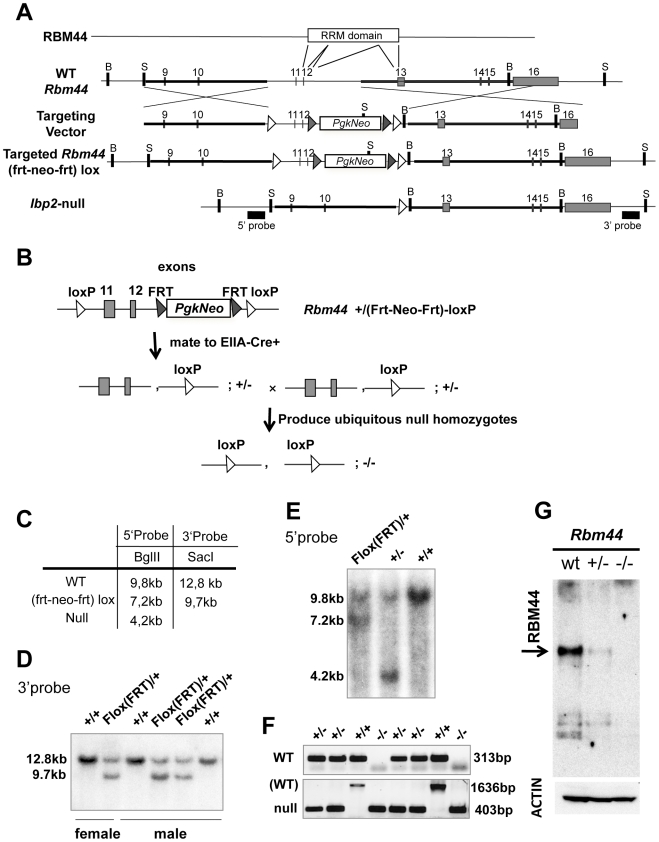
RBM44 targeted deletion *in vivo*. A, The strategy and conditional targeting vector to mutate the *Rbm44* locus in ES cells and delete the RRM domain in mice: open white rectangle, PGK1-neomycin expression cassette; black arrowhead, *frt* sequence; open white arrowhead, *loxP* sequence; B, *BglII* site; S, *SacI* site. Exons 11 and 12 are deleted in *Rbm44* null mice. Southern probes are shown as black boxes. B, The mating strategy to generate a ubiquitous deletion of RBM44. C, The strategy to detect *Rbm44* WT, targeted, and deleted alleles. D, Southern blot analysis using restriction enzyme SacI and 3′probe shows recombination of *Rbm44* in targeted ES cells (wild-type allele, 12.8 kb; targeted *Rbm44^frt-neo-frt–loxP^* allele, 9.7 kb). E, Southern blot analysis using restriction enzyme BglII and 5′probe shows targeted deletion of *Rbm44* in mice (wild-type allele, 9.8 kb; targeted *Rbm44^frt-neo-frt-loxP^* allele, 7.2 kb; *Rbm44* null allele, 4.2 kb). F, PCR genotyping of *Rbm44* null mice. (WT, 313 bp; WT in bottom panel, 1636 bp; null, 403 bp). G, Western blot analyses of total testis extracts of wild-type (WT), *Rbm44* heterozygous (*Rbm44^+/−^*), and null (*Rbm44^−/−^*) mice using anti-RBM44 antibody generated against amino acids 471–609 (N-terminal of the deletion) and anti-ACTIN antibody for a control.

**Figure 9:**
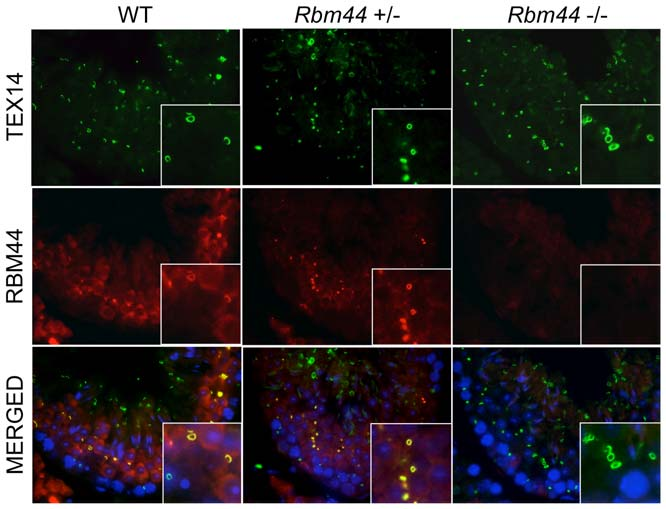
The RBM44 deleted mice have intercellular bridges. Immunofluorescence analysis of 10-week-old wild-type, *Rbm44* ^+/−^ and *Rbm44* ^−/−^ mice testes: green, TEX14; red, RBM44; blue, DAPI; yellow, merged. The white boxes show high magnification images. In these panels, the anti-RBM44 antibody was generated against amino acids 471–609 (N-terminal of the deletion).

To define the functions of RBM44 *in vivo*, we generated a targeted deletion of *Rbm44* in mice. Because the RRM domain in mouse *Rbm44* spans from exon 11 to exon 13 (Figure 8A), we designed a knockout strategy to delete this domain. The *Rbm44* conditional targeting vector has a single *loxP* sites in the intron between exons 10 and 11 and the *frt-pgkNeo-frt-loxP* sequence in the intron between exons 12 and 13. ES cells with a targeted *Rbm44* allele (*Rbm44* ^tm1Zuk^; herein called *Rbm44* ^(frt-neo-frt)-loxP^) were identified. The chimeric male mice (*Rbm44* ^+/(frt-neo-frt)-loxP^) were mated to *EIIA-Cre* female mice to produce *Rbm44* heterozygous mutant (*Rbm44* ^+/−^) mice, which were then intercrossed to generate *Rbm44* null mice (*Rbm44^−/−^*) (Figure 8B). Southern blot using 5′ and 3′probes (Figure 8A) and PCR were performed to genotype mice, and the targeted deletion of exon 11 and 12 was confirmed in mice (Figure 8C to F). After performing western blot analysis of total testis lysates, RBM44 protein was shown to be decreased in *Rbm44* heterozygous testes and absent in the *Rbm44* homozygous testes using an antibody generated against RBM44 amino acids 471–609 (i.e., N-terminal to the deletion)(Figure 8G). Immunofluorescense analyses of testes from wild-type, *Rbm44* heterozygous, and *Rbm44* null mice also showed RBM44,-negative/TEX14-positive intercellular bridges only in the *Rbm44,* ^−/−^ mice (Figure 9). The absence of expression of RBM44 by Western blot and immunofluorescence using an antibody N-terminal to the region deleted confirms that our mutation prevents the expression of RBM44 and confirms that the mutant allele is null.

### RBM44 is not required for fertility

**Figure 10:**
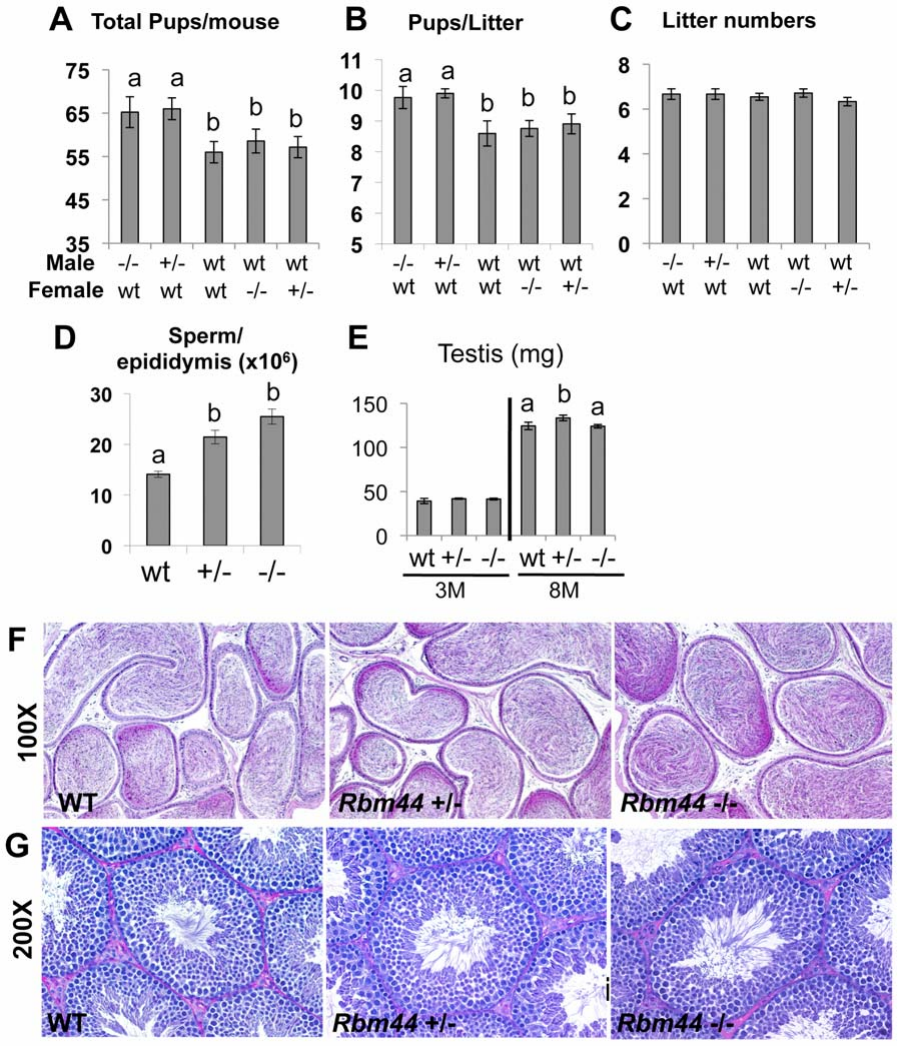
RBM44 deleted male mice are fertile. A, The number of total pups for 6 months, B, the number of pups per litter, and C, the total litters for 6 months are shown. A–C, the combinations of male: female used for mating are listed at the bottom of the graphs. male: female =  *Rbm44^−/−^*: WT, *Rbm44^+/−^:*WT, WT:WT, WT: *Rbm44^−/−^*, WT: *Rbm44^+/−^* (from left to right in each graph). D, Testes weights of 3-week-old and 8-month-old wild-type, *Rbm44^+/−^*, and *Rbm44^−/−^* mice were examined (a vs. b, p<0.05). E, the number of caudal epididymal sperm from wild-type, *Rbm44^+/−^*, and *Rbm44^−/−^* mice. F-G, PAS staining of 3-month-old testes (F), and 10-week-old caudal epididymides (G) in wild-type, *Rbm44^+/−^*, and *Rbm44^−/−^* mice.

The number of total pups per mouse and the number of pups per litter were slightly increased in *Rbm44* heterozygous and null males when they crossed with wild-type females (Figure 10A and B). However, the litter numbers of *Rbm44* heterozygous and null male were as frequent as wild-type males (Figure 10C). There were no significant differences in fertility between *Rbm44* heterozygous and null males (Figure S1). On the other hand, female fertility was not different among wild-type and *Rbm44* heterozygous, and *Rbm44* homozygous mutant mice. (Figure 10A–C and Figure S2).

### The deletion of RBM44 leads to an increase in number of sperm

Caudal epididymides were dissected from 3-month-old wild-type, *Rbm44* heterozygous, and *Rbm44* null male mice. Sperm were collected from the caudal epididymides and counted. The number of sperm of both *Rbm44* heterozygous and null male were significantly increased more than that of wild-type males, although there were no significant increases in the sperm counts between *Rbm44* heterozygous and null males (Figure 10D). Cauda epididymides *of Rbm44* heterozygous and null mice were more concentrated with sperm than the caudal epididymides of wild-type mice, which were processed at the same time and manner from dissection through PAS-hematoxylin staining (Figure 10F). In contrast, there was no significant differences in the histology of wild-type, *Rbm44* heterozygous, and *Rbm44* homozygous mutant testes (Figure 10E and G).

## Discussion

We previously reported that the lack of intercellular bridges in TEX14 null male mice results in the failure of spermatogenesis and infertility [9]. Our study showed that intercellular bridges are essential and there is massive spermatocyte apoptosis in *Tex14*-null testis. Cytoplasmic contiguity maintained by intercellular bridges may ensure that cytoplasmic components, especially mRNAs, freely diffuse between interconnected cells. Because RBM44 carries an RNA recognition motif (RRM) and the RRM could potentially bind a multitude of RNA sequences and proteins (reviewed in reference [29]), we suspect that RBM44 may function as an RNA-binding protein required for intercellular bridge function. We found that RBM44 interacts with itself and TEX14 using yeast and mammalian two-hybrid analyses. Unlike TEX14, RBM44 does not function in the formation of stable intercellular bridges. The presence of abundant cytoplasmic RBM44, in addition to its specific enrichment at intercellular bridges in the spermatocytes, led us to suspect that RBM44 migrates from the cytoplasm to the intercellular bridges during meiosis, integrates into the bridge, and then becomes dispersed from the bridges after round spermatids are formed. The RRM of RBM44 might bind to RNAs in the cytoplasm and help to shuttle them through the intercellular bridge, facilitating their dispersion into the interconnected neighboring cells. However, this hypothetical function of RBM44 is indispensable for intercellular bridge formation or function or male fertility, and no discernible histological differences were observed in the testes of wild-type and *Rbm44* null mice. Because *TB-RBP*-null male mice had abnormal seminiferous tubules and reduced sperm counts but were fertile, it is possible that male germ cells contain redundant pathways to regulate the transport and temporal expression of post-transcriptionally regulated mRNAs [22]. For example, BRUNOL1 (also known as CELF3), a brain-testis specific RNA binding protein, is also expressed in the haploid stage of spermatogenesis. Even though the targeted deletion of BRUNOL1 reduced spermatogenesis, there is no significant abnormality in testis and knockout mice was fertile [30]. Thus, multiple systems may exist to regulate post-transcriptional germ cell functions such as mRNA transport through intercellular bridges (if it is regulated) and translation. It remains to be seen if RBM44 is redundant with other RNA-binding proteins.

## Supporting Information

Figure S1 ***Rbm44*** **heterozygous (** ***Rbm44^+/−^*** **) and null (** ***Rbm44^−/−^*** **) male mice have similar fertility.** *Rbm44^+/−^* and *Rbm44^−/−^* male were mated with wild-type female mice. The 6 month mean number of total pups per month per female (A), mean litter size (B), litters/month (C), days between deliveries (D) between *Rbm44^+/−^* and *Rbm44^−/−^* male mice, and details of A–D are shown (E).
(TIF)

Figure S2 **There is no differences in fertility between** ***Rbm44*** **heterozygous (** ***Rbm44^+/−^*** **) and null (** ***Rbm44^−/−^*** **) females.** Fertility indexes of *Rbm44^+/−^* and *Rbm44^−/−^* females by mating with wild-type male are shown. A, The 6 month mean number of total pups per month per female. B, Mean litter size. C, Litters per month. D, Days between deliveries. E, Detail of A–D.
(TIF)

Figure S3 **Staging of RBM44 expression in testis.** Immunohistochemistry in 3-month-old mice testis using anti-RBM44 antibody was examined by the staging of spermatogenesis in comparison with PAS staining in serial sections.
(TIF)
