# Supplementary figures and images for: Identification and Characterization of RBM44 as a Novel Intercellular Bridge Protein
Source: PLoS One. 2011 Feb 25;6(2):e17066. doi: 10.1371/journal.pone.0017066 (PMC3045441; doi:10.1371/journal.pone.0017066)

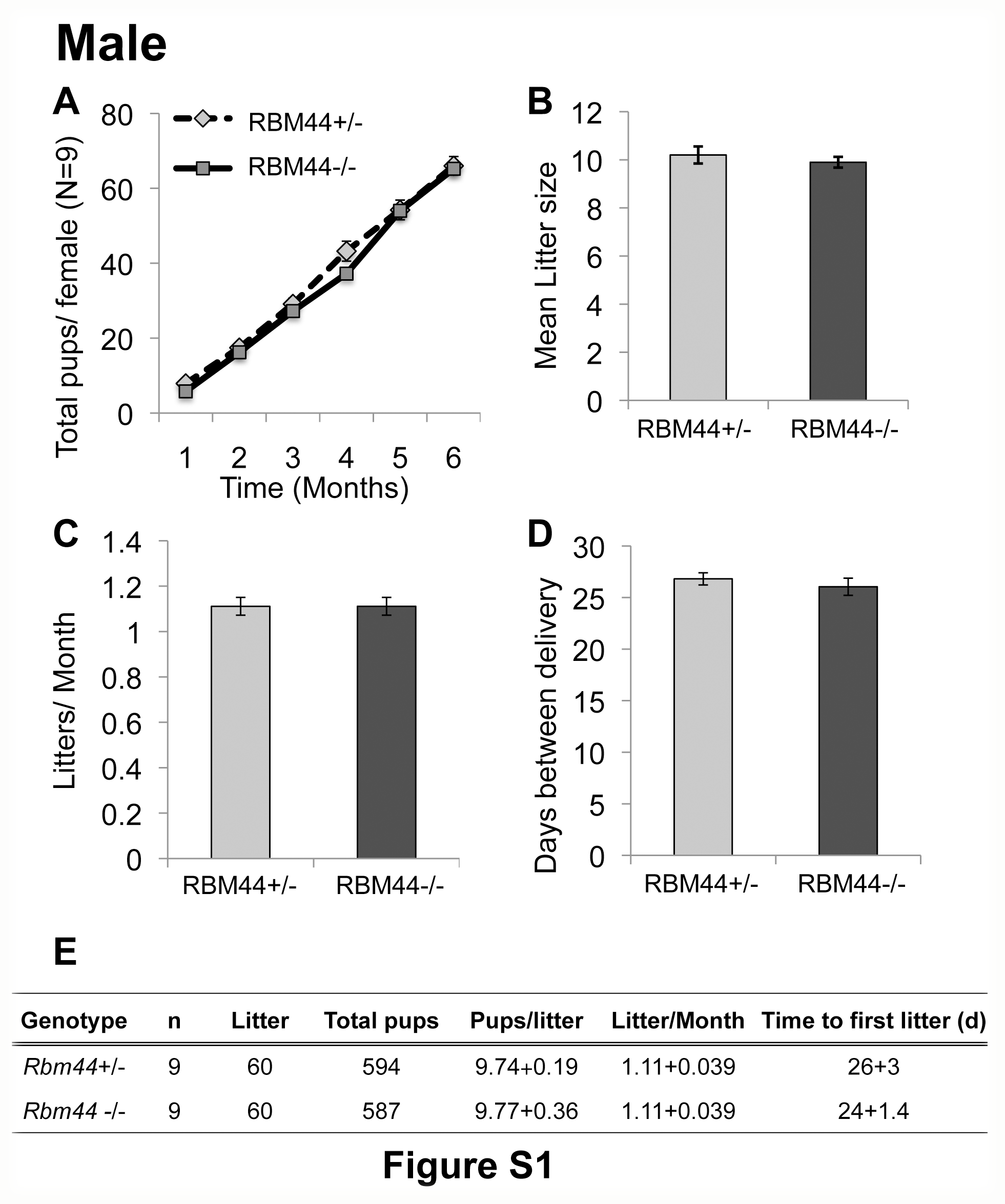

Supplement: Figure S1 — Rbm44 heterozygous ( Rbm44+/− ) and null ( Rbm44−/− ) male mice have similar fertility. Rbm44+/− and Rbm44−/− male were mated with wild-type female mice. The 6 month mean number of total pups per month per female (A), mean litter size (B), litters/month (C), days between deliveries (D) between Rbm44+/− and Rbm44−/− male mice, and details of A–D are shown (E). (TIF) [file pone.0017066.s001.tif]

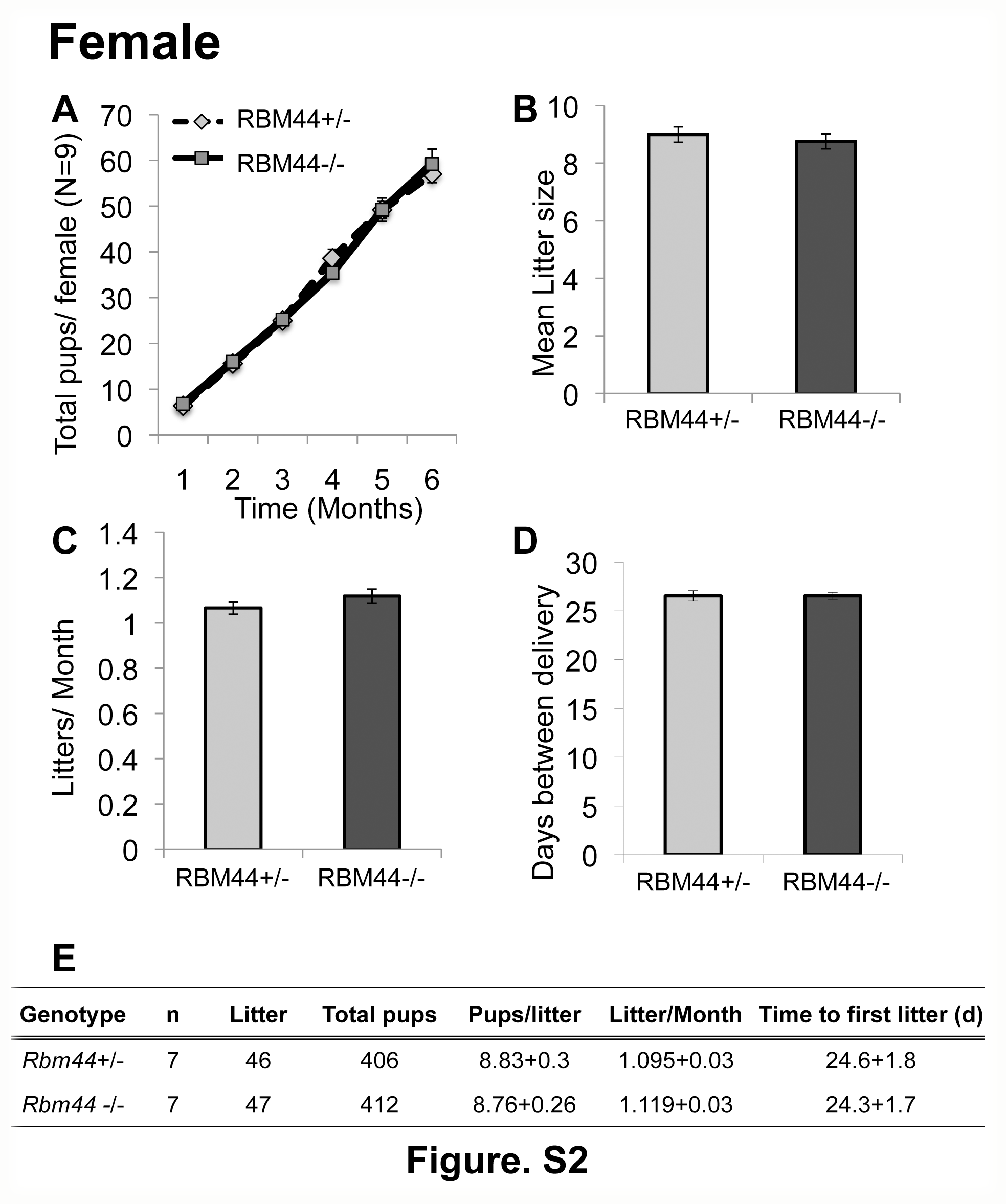

Supplement: Figure S2 — There is no differences in fertility between Rbm44 heterozygous ( Rbm44+/− ) and null ( Rbm44−/− ) females. Fertility indexes of Rbm44+/− and Rbm44−/− females by mating with wild-type male are shown. A, The 6 month mean number of total pups per month per female. B, Mean litter size. C, Litters per month. D, Days between deliveries. E, Detail of A–D. (TIF) [file pone.0017066.s002.tif]

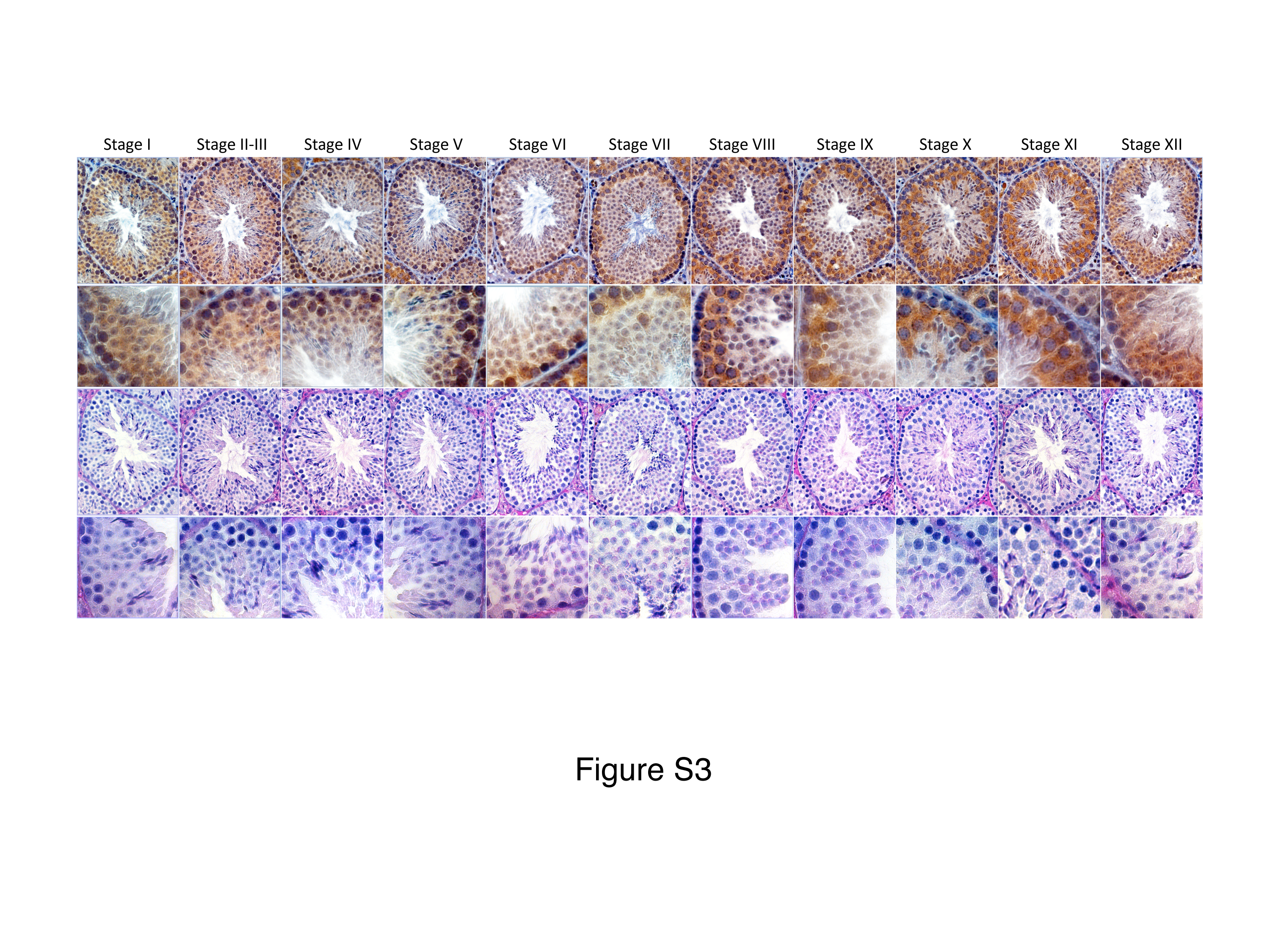

Supplement: Figure S3 — Staging of RBM44 expression in testis. Immunohistochemistry in 3-month-old mice testis using anti-RBM44 antibody was examined by the staging of spermatogenesis in comparison with PAS staining in serial sections. (TIF) [file pone.0017066.s003.tif]
